# Factors Impacting Patient Outcomes Associated with Use of Emergency Medical Services Operating in Urban Versus Rural Areas: A Systematic Review

**DOI:** 10.3390/ijerph16101728

**Published:** 2019-05-16

**Authors:** Ahmed Ramdan M. Alanazy, Stuart Wark, John Fraser, Amanda Nagle

**Affiliations:** School of Rural Medicine, University of New England, Armidale NSW 2351, Australia; aalanazy@myune.edu.au (A.R.M.A.); jfrase22@une.edu.au (J.F.); anagle2@une.edu.au (A.N.)

**Keywords:** emergency medical services, rural, urban, patient outcome, survival rate

## Abstract

The goal of this systematic review was to examine the existing literature base regarding the factors impacting patient outcomes associated with use of emergency medical services (EMS) operating in urban versus rural areas. A specific subfocus on low and lower-middle-income countries was planned but acknowledged in advance as being potentially limited by a lack of available data. Preferred Reporting Items for Systematic Reviews and Meta-Analyses (PRISMA) guidelines were followed during the preparation of this systematic review. A comprehensive literature search of PubMed, EBSCO (Elton B. Stephens Company) host, Web of Science, ProQuest, Embase, and Scopus was conducted through May 2018. To appraise the quality of the included papers, the Critical Appraisal Skills Programme Checklists (CASP) were used. Thirty-one relevant and appropriate studies were identified; however, only one study from a low or lower-middle-income country was located. The research indicated that EMS in urban areas are more likely to have shorter prehospital times, response times, on-scene times, and transport times when compared to EMS operating in rural areas. Additionally, urban patients with out-of-hospital cardiac arrest or trauma were found to have higher survival rates than rural patients. EMS in urban areas were generally associated with improved performance measures in key areas and associated higher survival rates than those in rural areas. These findings indicate that reducing key differences between rural and urban settings is a key factor in improving trauma patient survival rates. More research in rural areas is required to better understand the factors which can predict these differences and underpin improvements. The lack of research in this area is particularly evident in low- and lower-middle-income countries.

## 1. Introduction

Traumatic injuries are one of the leading causes of death around the world, with the World Health Organization (WHO) [1] noting that the estimated five million traumatic injury-related deaths annually was equivalent to the combined deaths associated with human immunodeficiency virus (HIV)/acquired immunodeficiency syndrome (AIDS), malaria and tuberculosis. It is known that one of the most significant factors affecting health outcomes in trauma patients is time [2], with the period before commencement of healthcare support and then while transferring a patient from the original incident site to a health service considered critical to reducing mortality and improving recovery for the individual [3]. The concept of a ‘golden hour’, the length of time immediately after the trauma and until care is provided, is often used as a key performance measure for emergency medical care, although the validity of the implied ‘60 minutes’ timeframe is open to question [4]. However, the potential magnitude of any gap between low-, middle- and high-income countries [5] in regard to the timely and effective provision of care by emergency medical services (EMS), and particularly in relation to rural versus urban differences, has not been comprehensively explored. 

EMS provide health assistance to patients affected by sudden illness or injury [6] and are often the first line of care responders for individuals during medical emergencies. The roles of EMS include providing primary on-site emergency healthcare, ongoing care during transportation from the location, and then the transfer of care upon arrival at the nearest appropriate receiving healthcare facility [7]. As an example of the importance of an effective EMS, it has been estimated that 74% of ischemic heart disease mortality occurs either outside of, or prior to, a patient’s arrival at a hospital, and therefore, that any prehospital care provided by support options plays a vital role in increasing survival rates [8]. Similarly, patients with coronary artery diseases and trauma were found to have higher levels of preventable deaths due to the delayed receipt of prehospital care by an EMS [9]. Factors that may limit the ability and capacity of an EMS to provide appropriate prehospital care include the geography of the area, equipment availability and training in life-saving protocols [10].

The association between response time interval, the period between notification of an incident and EMS arriving on the scene, and improved survival rates has been strongly established and has subsequently become an international standard for EMS, particularly in urban settings [11]. Evidence from the US National Academy of Sciences and National Research Council, which described the proportions of mortality and morbidity related to car accidents as well as the severe deficiencies of the delivery of prehospital care [12], underpinned the development of the USA 1973 Emergency Medical Services (EMS) Systems Act (PL 93–154). However, while the EMS Act determined many criteria in emergency service provision, it made no recommendations regarding what an acceptable response time interval should be [13]. 

Prehospital intervals refer to the time between an incident occurring and the patient arrival at the nearest appropriate health facility. As a consequence of the significant differences that are inherent both within and between different countries, attempts to define target goals for prehospital intervals have remained difficult. One recommendation for a prehospital interval time noted that patients should receive basic life support within four min and advanced life support within eight min of a traumatic event [14], but this goal was recognised as being impossible to achieve in some rural locations. A National Association of EMS Physicians position paper reported that response and transport time intervals vary according to the region and thus should be locally determined but offered no definitive standards for EMS to meet in rural or urban areas [15]. 

The existing literature differs regarding the timeframes implied by the term ‘golden hour’ in response to injury [16]. In addition, debate exists regarding the grade at which the victims would be treated by health professionals at the site of the trauma before transferring to a hospital. The American College of Surgeons Advanced Trauma Life Support recommends quick transport to a trauma centre and call specialist to reduce on-scene time [17], but this approach is not followed in all countries. Therefore, establishing the association between prehospital interval and mortality outcomes is complicated, as in some countries, paramedics are supported by medical professionals on-site in starting life-saving treatment before transfer, while in other countries, only paramedics attend the trauma site and work towards immediately transferring the patient to a hospital. 

Urban–rural difference in the performance of EMS in achieving better patient outcomes is an issue of limited but ongoing investigation in high-income countries [18]. As an example, Jennings, Cameron, Walker, Bernard and Smith [19] found that cardiac patients in an urban area had significantly higher survival rates than patients in rural areas; this difference was predominantly ascribed to the time differential in EMS arrival on-scene. Studies on EMS in the USA revealed a substantial difference in response and transfer times between the urban and rural models [20,21]. Similar findings were noted by Aftyka, Rybojad and Rudnicka-Drozak [22] in Poland, who found that EMS in a rural area had a significantly longer response time than in an urban location. 

This problem is partially explained by the disparate geographic distances involved; in one study, the urban EMS had to travel less than ten kilometers to reach the incident site, while in rural locations, over a third of responses were more than thirty kilometers from the EMS starting point [22]. However, simple distance is not the only reported factor for potential differences in response time. Other noted obstacles include a declining number of emergency departments, availability and/or deficiency of appropriate vehicles, the insufficiency of roads and incapability to pay for transport services [7,16,23].

The purpose of this current systematic review was to examine the literature base regarding urban versus rural differences in EMS in relation to patient outcomes. Specifically, the overall goal was a focus on the identification and review of all such research in low- and lower-middle-income countries, as it has been noted by the United Nations (UN) that, while nearly half the world’s population live outside of a metropolitan area, this proportion is generally even larger in countries with less developed infrastructure [24]. However, it was recognised that the existing literature in this field might be limited, and therefore, it was predicted likely to have to encompass findings on urban versus rural differences in EMS patient outcomes from all countries. The overarching review question was defined as “What factors differentiate urban and rural EMS and contribute to differences in patient outcomes in low- and lower-middle-income countries?” 

## 2. Methodology

### 2.1. Definition of Rural Versus Urban

Prior to commencement of the systematic review, the definition of what constituted ‘rural’ versus ‘urban’ needed to be determined. Throughout both the literature base and government sites, definitions of rural and urban are inconsistent and vary dramatically from one country to another, and even within countries. The United Nations Statistics Division [25] has recognised this problem and argued that “the distinction between urban and rural population is not amenable to a single definition applicable to all countries. For this reason, each country should decide which areas are to be classified as urban and which as rural, in accordance with their own circumstances”.

The decision was made to follow the definition used in each separate research paper, and it is acknowledged that this would result in differences of definition across the entirety of the articles. However, it was felt that this compromise was the most appropriate solution as each paper would have used the relevant definition for that specific location. Any attempt to re-analyse all of the available data to conform to one definition, if such a definition could even be agreed upon, was deemed beyond the scope of this review.

### 2.2. Systematic Review Protocol 

While the overall goal was to identify all relevant literature in lower- and lower-middle countries, it was predetermined to commence the search without this restriction. It was decided in advance that this additional stage of screening could be a final step in the protocol if at least 30 articles solely from lower- and lower-middle countries were located; otherwise, all relevant papers would be included irregardless of the country of origin.

The Preferred Reporting Items for Systematic Reviews and Meta-Analyses (PRISMA) guidelines were used to identify all relevant papers that reported on trauma patient outcomes between rural and urban EMS [26]. All steps were performed in accordance with the Cochrane Handbook of Systematic Review [27]. A specialist health research librarian provided assistance in developing the relevant terms and in undertaking the search. To query the databases, an electronic search of PubMed, EBSCO (Elton B. Stephens Company) host, Web of Science, ProQuest, Embase, and Scopus was performed in May 2018 using the following keywords: (“Emergency Medicine” OR “Emergency Service” OR “Emergency Medical Services” OR “Emergency Medical Technicians” OR “paramedic*” OR “emergency” OR “trauma” OR “ambulance*” OR “pre-hospital” OR “out of hospital” OR “EMT” AND “Urban Population” OR “Hospitals, Urban” OR “Urban Health Services” OR “Urban Areas” OR “Urban Health” OR “Rural Health Personnel” OR “Hospitals, Rural” OR “Rural Population” OR “Rural Health Services” OR “Rural Areas” OR “Rural Health Centres” OR “Rural Health” AND “Patient-Reported Outcomes” OR “Outcomes (Health Care)” OR “Treatment Outcomes” OR “patient outcomes” OR “patient progress” OR “patient*” AND “Survival Rate” OR “Response Time” OR “Golden Hour”). 

The search for relevant literature was limited to articles published in English within the last 30 years (1988 to 2018). The initial search found a total of 2235 articles; this was composed of 134 articles identified in PubMed, 214 in EBSCOhost. 340 in ProQuest, 246 in Embase, 472 in Scopus, and 829 in Web of Science.

### 2.3. Screening

**Figure 1:**
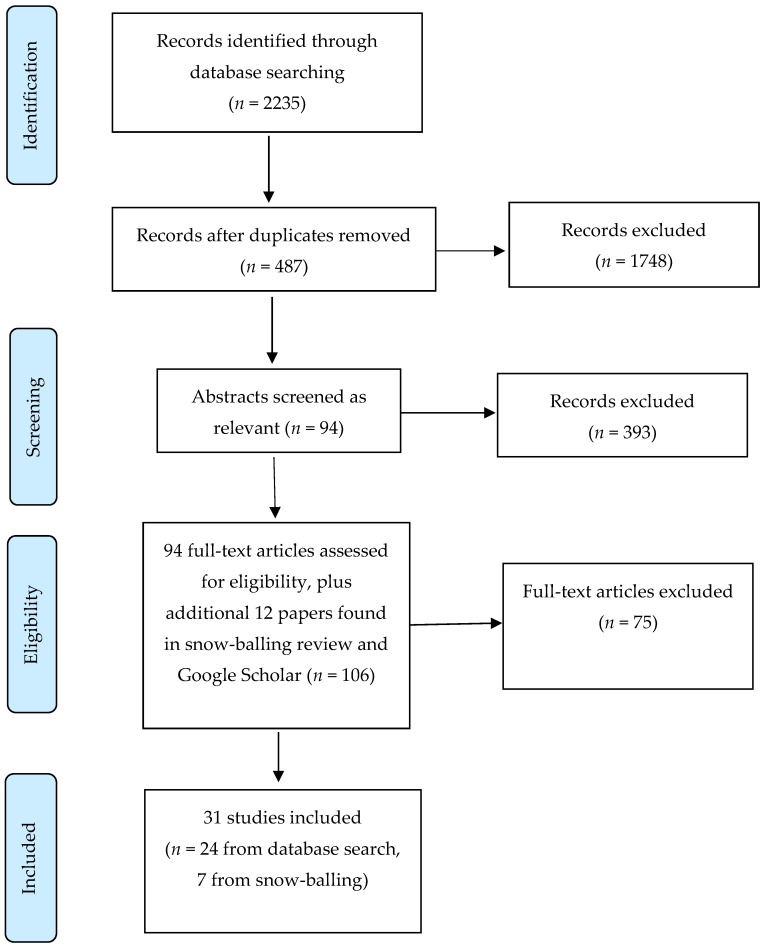
The Preferred Reporting Items for Systematic Reviews and Meta-Analyses (PRISMA) flowchart of the article selection.

The two lead authors independently screened the literature search output to identify relevant studies. The eligibility screening was performed in sequential stages, as outlined in Figure 1 below. After identifying and removing duplicates within the originally identified sample of 2235, the next stage was to screen titles and abstracts of the 487 remaining records and to remove any articles that were not related to the defined topic area. This was undertaken by each of the two authors separately, and the results then compared. It was again predetermined that any differences of opinion regarding the inclusion or exclusion of articles would be discussed and consensus reached. If consensus could not be reached, a third author agreed to act as the independent reviewer. However, this was ultimately not needed at any stage.

The next step saw each author evaluate the full text of 94 articles identified in the previous stage as being potentially relevant. Of these 94 papers, 24 were deemed as relevant after review (see Table 1). A process of ‘snow-balling’ was also performed at this point by reviewing the reference lists of each paper for any additional articles that may be relevant. Another 12 articles were identified, of which 7 were included. Finally, a check of Google Scholar was undertaken to check that no other potential manuscripts were available, but no additional papers were found. In total, 31 articles were deemed as eligible for inclusion. 

As noted earlier, screening for papers solely from low- and lower-middle-income countries was planned to occur as a final stage. However, a preliminary review of the results indicated that only one study, from India, was found to be from a low- or lower-middle-income country. As this would not culminate in the predetermined figure of 30 articles, the proposed final stage of screening did not occur, and all articles from all countries were included. Forty two percent of the research was undertaken in the USA (*n* = 13). Approximately 20% of the studies were from the Nordic countries of Sweden, Norway, Denmark and Finland, while Australia, the UK and Ireland provided another 23% (*n* = 3, 3 and 1, respectively). 

### 2.4. Quality Appraisal

To determine whether any of the 31 studies should be excluded on the basis of quality, the Critical Appraisal Skills Programme (CASP) model was employed [28]. This assessment process examined each paper on three broad issues: Are the results of the study valid?; What are the results?; and Will the results help locally?, and each study could get a maximum score of 12 [29]. All 31 articles were analysed by the two lead authors to gain a better understanding of the objectives, themes and major findings of each paper. A summary of each of the 31 papers, along with the results of the quality appraisal, are presented chronologically (1991 to 2018) in Table 1 below; no papers were evaluated as being of overall poor quality and therefore requiring removal.

## 3. Results

The following Results section is organised by key themes arising from analysis of the 31 articles.

### 3.1. Prehospital Time

Four studies reported on prehospital time interval. The results showed that prehospital time was significantly shorter in urban locations than in rural areas. Gonzalez et al. [29] reported that prehospital time in rural locations was 42.0 min, while it was 24.8 min in urban areas (*p* < 0.0001). McGuffie et al. [37] found that all prehospital times were significantly longer for rural patients than those in urban settings (*p* < 0.001). Nordberg et al. [50] showed that median time to arrival varied between 0.8 to 3.2 min in urban and rural areas but that rural areas had longer arrival times. Raatiniemi et al. [51] noted that rural areas had longer prehospital times and also noted, not surprisingly, that there were longer geographic distances to travel than urban areas.

### 3.2. Response Time

Differences between rural and urban areas in terms of response time were found across nine studies. Aftyka et al. [22] reported that interventions in the urban areas were associated with a significantly lower response time than the rural areas, as did Moore et al. [39] and Vukmir [36]. The results by Masterson et al. [49] revealed that urban patients received an EMS response on average eight min more quickly than rural patients (33% vs. 9%; *p* < 0.001). Sørensen et al. [43] noted that rural areas had longer geographic distances to travel (median of 30 km) than urban areas, and this resulted in an average response time of nine min more in rural than urban areas (*p* < 0.001). 

Gonzalez et al. [20] reported a response time of 13.9 min (rural) vs. 11.2 min (urban) (*p* < 0.0002), and this resulted in increased mortality rates in the rural settings. The study by Gonzalez et al. [29] found mean response times of 10.67 versus 6.50 min in rural and urban settings, respectively (*p* < 0.0001). Grossman et al. [32] again highlighted a discrepancy between locations, with mean response times of 13.6 min (urban) and 7 min (rural) (*p* < 0.0001). In addition, Layon et al. [34] showed more rapid responses to patients in the city than in the country. However, it is worth noting that not all studies noted a difference. Stripe et al. [30] reported that the rural and urban areas response times were equivalent, but only after excluding the rural area long-distance transfers.

### 3.3. On-scene Time

Three studies reported on on-scene time—the period between arrival at the location and either the resolution of the issue or transportation to another site commencing. These studies argued that on-scene time was significantly shorter in urban than rural settings. Gonzalez et al. [20] reported that mean rural EMS time on scene was 16.1 min, which was significantly higher than for urban settings (11.6 min). Gonzalez et al. [29] noted that the EMS scene time in urban settings was 10.83 min, versus 18.87 min for rural settings (*p* < 0.0001). Grossman et al. [32] found that the mean scene time in urban areas was again lower than in rural areas (18.7 vs. 21.7 min).

### 3.4. Transfer Rates

Two studies reported on transfer rates, which is the proportion of patients who required transportation from the scene to a health facility. Newgard et al. [21] reported that rural areas had longer transfer distances and higher transfer rates (3.2% vs. 2.7%). This finding was in contrast to the results of Horeczko et al. [46], who had found that urban and rural emergency departments showed similar transfer rates.

### 3.5. Transport Time

Four studies reported on transport time—the period for transportation from the scene of the incident to the nearest appropriate health facility—and they showed significantly shorter transport times in urban than rural settings. Fatovich et al. [42] found that mean transport times to definitive care were 59 min in urban settings versus 11.6 h in rural settings (*p* < 0.0001), although it is worth noting that a possible increase in the usage of emergency air services in recent years in Western Australia may have at least partially reduced this extremely large difference. Gonzalez et al. [29] noted a mean transport time was 12.45 min in rural versus 7.43 min in urban areas (*p* < 0.0001). Vukmir [36] reported that transport times were decreased in suburban sites compared to rural sites. Grossman et al. [32] found that mean transport times from the scene to the hospital were 8.2 min in urban versus 17.2 min in rural areas (*p* < 0.0001).

### 3.6. Survival Rates

Seventeen studies reported that EMS patients living in urban areas had higher survival rates than those in rural areas. A study by Park et al. [54] showed that good neurological recovery was demonstrated in 1.6% versus 6.8% of the patients in rural and urban areas, respectively (*p* < 0.01). Mathiesen et al. [53] reported that urban patients had higher survival chance to hospitals admission (odds ratio: 1.58, 95% CI 1.11–2.26, *p* = 0.012) than rural patients. Nordberg et al. [50] found that 30-day survival was higher in urban patients than in rural patients. Research by Masterson et al. [49] noted that urban patients were also more likely to be discharged alive from hospitals than rural patients. Similarly, Fatovich et al. [42] reported that there was a significantly increased risk of death in rural areas than in urban areas (OR 2.60, 95% CI 1.05–6.53, *p* = 0.039). Beck et al. [52] found that cardiac arrests occurring in rural regions had significantly lower odds of attempted resuscitation relative to those in urban regions. Shultis et al. [41] reported a higher stroke death rate in rural areas than in urban areas. Grossman et al. [32] found that rural patients had a higher risk of death before arrival (relative risk = 7.4) when the response time was over 30 min. Bhuyan et al. [44] reported that patients in urban areas had a lower risk of death from acute myocardial infarction than patients in rural areas. Jennings et al. [19] and Ro et al. [45] also showed higher survival rates in urban than in rural areas.

It is worth noting that these findings of reduced survival rates for rural residents were not consistent across all the papers, with three papers reporting the opposite trend. A study by Raatiniemi et al. [51] reported that mortality within 30 days was 23.9% and 13.3% in urban and rural settings, respectively. Mihalicz et al. [40] found that urban patients had a higher death rate than rural ones (13.0% vs. 10.5%; *p* = 0.05). Another study by Lombardi et al. [31] showed that survival was significantly lower in urban than in suburban/rural areas. Other studies showed that urban and rural EMS were comparable regarding their survival rates [21,32,38,47].

## 4. Discussion

The purpose of the current study was to investigate the factors that lead to differences between EMS in rural and urban areas, and specifically in relation to patient outcomes. Initially, the goal was to examine any potential differences solely within low- and lower-middle-income countries [5]. However, it became clear that the literature base was not sufficient to support this approach, and therefore, research from all countries was included. The results indicated some reasonably consistent observed differences between rural and urban EMS. Generally, urban EMS were associated with faster response time, less on-scene time, lower prehospital time intervals, lower transfer rates and reduced transport time, and urban patients had higher survival rates than rural patients. It is acknowledged that, particularly in rural areas, the reasons for any observed differences between rural and urban areas are often complicated and associated with multilevel problems, and there was considerable diversity in the results between countries.

### 4.1. Response Time

The results showed that, generally, urban areas had a shorter response time than rural areas. This is not surprising, as due primarily to geographic distance, EMS simply take substantially longer to arrive on the scene in rural areas. Other factors that have been identified as affecting response time in rural regions include the number and location of emergency medical services, the type and number of ambulances, the physical condition and maintenance of roads and transport infrastructure and an inability of poorer rural residents to pay for transport services [7,20,49]. The response time interval is dependent on the distance to the incident and the maximal speed at which ambulances can safely travel, including roadway conditions and traffic. A differential of nearly 50% was noted for response times between rural and urban areas, with Aftyka et al. [22] identifying that the average response time should not exceed eight min in urban regions and fifteen min in rural regions, while the third quartile of response time should be no more than 12 min in urban regions and 20 min in rural regions. 

There have been some attempts to reduce response times, with an example being the National Ambulance Service in South Korea, which has started to dispatch non-frontline officers and moderate care ambulances to specific situations, such as cardiac arrest calls, which may not require the same levels of responder training as more complicated road accident scenarios [45]. Nonetheless, it is clear that there is still a disparity between rural and urban areas in terms of response time. It is not possible to overcome some of the barriers associated solely with geographic distance, as it is clearly not economically viable to have specialist care located in every small rural village, but a mapping of need against service access may assist in identifying where new services could be established, or where existing undersubscribed services could be relocated.

### 4.2. On-Scene Time

While disparities regarding response time are somewhat understandable as a consequence of geographic distance, the results showed that the on-scene time was also generally shorter in urban than in rural locations. The reasons for this difference are not as clear-cut as with response time. It is known that on-scene time is affected by the choice of the initial stabilising method, the number of responders and the time required for safely preparing the patient for transport. Other issues that were found to prolong on-scene time included increasing illness severity, a requirement for use of advanced intravenous devices, the mode of transport and complexity of traumatic cases [7,20,49]. However, these identified factors do not completely explain why there should be the observed differences between rural and urban locations. 

It is hypothesised that one factor that may contribute to the observed difference is the increased likelihood of severe traumas in rural areas, such as those associated with high-speed vehicle accidents and workplace injuries in the farming or mining sectors. Greater complexity in the presenting medical issues will result in increased on-scene time as the responders have to take greater care in stabilising and preparing the patient for transportation. Further, the use of a tiered system, where both a paramedic and an emergency life support team (ELST) are dispatched simultaneously to a callout, may also contribute towards the on-scene time interval. As an example of their role, an ELST (known by different names around the world) is normally licensed to perform more advanced interventions, such as inserting airway devices or intravenous lines for cardiac arrest patients. However, particularly in rural areas, not all paramedic centres will also have a co-located ELST, and smaller locations with paramedic service may instead be supported by an ELST situated in a larger regional or capital city. Therefore, the paramedic initial responders in rural areas who are awaiting the arrival of an ELST from another location will have to spend more time on-scene, whereas in urban settings, the joint arrival of both paramedics and ELST from one location will minimise any time differential. 

### 4.3. Transport Time Interval

As with response time, geographic distribution naturally also plays a vital role in the transport time interval. The results indicated that rural transport times were significantly longer than urban transport times. Distance is clearly one of the primary factors leading to this finding; however, other noted issues include the available transport options (e.g., air services were available to supplement road services), the road conditions, and traffic congestion. Many of these issues are already noted with respect to response time. However, it is worth recognising that transport time and response time are not necessarily the same in rural areas. This is due to the fact many rural trauma patients may not be transported to the nearest hospital and are instead sent to a specialist trauma centre. This is perceived to happen primarily when patients require a level of expert care that is not likely to be found in local non-trauma hospitals (e.g., [55]). In an urban setting, this issue does not appear to cause the same level of problem, as it is more likely that there is a trauma centre in similar proximity to that of the nearest hospital. The increasing use of air transport (i.e., helicopters, planes, etc.) in rural and remote areas has potential to decrease this difference, but this solution is both expensive and reliant on additional training and resources that are not viable in many areas of the world.

### 4.4. Survival Rates

The reviewed articles indicated that patients living in rural areas had lower survival rates from equivalent conditions when compared to those living in urban areas. Many of the issues associated with this differential in survival rates have been outlined above, with it argued that the difference is primarily due to the increased time taken for an EMS to arrive at the scene. Clearly, there are conditions that are very time-dependent with respect to outcome, with examples cited including the need for endotracheal intubation [55] and the presence of asystole or pulseless electrical activity [19]. Studies (e.g., [20,43]) indicated that, on average, for some specific conditions, urban residents started to receive life-saving interventions within the recommended timeframe, while commencement of interventions for rural patients fell outside the timeframe. Reducing the time to receiving support from an emergency department cardiopulmonary resuscitation (CPR) team was shown to increase survival rates [34], while increased time to commencement of advanced cardiac life support was associated with a 39% reduction in survival rates in rural areas [49]. These differences may partially explain observed variations in survival rates between urban and rural patients. 

### 4.5. Limitations of This Review

The current study has some limitations that may limit the generalisability of the findings. Firstly, while the goal was to focus on urban and rural differences specifically in lower- and lower-middle-income countries, there was simply not enough research to support this approach. Therefore, articles from anywhere in the world were included in the final review. This has meant that there are considerable variations in both geographical diversity and population bases in the included studies. Secondly, the inherent differences in care delivery across a wide range of diverse countries and settings made global averages difficult to determine for any process of comparison. Thirdly, as noted, there was an absence of uniformity in the reporting of prehospital care time. These data variations made comparing and compiling the quality of included studies challenging. Readers are encouraged to consider their own local service environment when considering the relevance of the following conclusions and recommendations. 

## 5. Conclusions 

This systematic review of the available literature indicates that EMS in urban areas are associated with lower prehospital time, response time, on-scene time and transport time in comparison to EMS in rural areas. The research base also indicated that survival rates were higher for patients living in a city location than for those residing in rural areas. However, there is only limited research on these issues, and the findings identified across different countries show considerable discrepancies. The review also found that there was almost no research in lower- and lower-middle-income countries, with only one study that reported on this area. The potential for service discrepancies between rural and urban settings is arguably even higher in low-income countries, and as such, the findings of this literature review may not accurately capture the actual level of any problems in many parts of the world. As noted in the Limitations section, this level of disparity emphasises that the findings cannot be generalised, and the reader should interpret the results in light of their specific circumstances.

One of the key differences between rural and urban EMS related to on-scene time, and the increased time in rural locations could not be attributed solely to geographic isolation. It is possible that EMS require additional time on-scene in rural areas as a consequence of the longer response time, but this was not clear, and other unexplored factors could be evident. It is recommended that this issue should be subject to specific future consideration, with the level and appropriateness of training of staff, the timely availability of key support services such as ELST and the severity of patient injury three potential key research foci in this area. Further research that specifically examines approaches that will facilitate an improvement in rural areas, be it through changes to emergency medicine facilities, tools, vehicles or personnel, including tailored training, in order to provide a fast and efficient response to emergency situations on-site, is also recommended. 

## Figures and Tables

**Table 1:** Overview and quality assessment of articles.

Author & Year	Study Title	Study Design	Setting & Date (When Stated)	Patient Age Demographics	Study Sample	Outcomes of Interest	CASP Grade
Stripe and Suaman 1991 [30]	A rural-urban comparison of prehospital emergency medical services in Nebraska	Prospective study	The USA. 1988–1989	Not specified	Patients attending emergency medical services	The rural ambulance provided more services than an urban ambulance.	5/12
Lombardi et al. 1994 [31]	The outcome of out-of-hospital cardiac arrest in New York City. The Pre-Hospital Arrest Survival Evaluation (PHASE) Study	Cohort study	The USA 1990–1991	70 (30–79)	3243 patients with Cardiac arrest	Total survival rate within the study group was 1.4%.	6/12
Grossman et al. 1997 [32]	Urban-rural differences in the prehospital care of major trauma	Prospective cohort study	The USA. 1991–1992	Not specified	459 patients with major trauma	Rural patients had a higher risk of death before arrival (relative risk = 7.4, 95% Confidence Interval (CI) 2.4–22.8) if response time was over 30 min.	8/12
Absalom et al. 1998 [10]	Out-of-hospital cardiac arrests in an urban/rural area during 1991 and 1996: Have emergency medical service changes improved outcome?	Retrospective analysis	The UK 1991–1996	67 (13)/70 (13)	113 cases of out-of-hospital cardiac arrest	Restoration of spontaneous circulation before arrival in the Emergency Department (ED) occurred for patients irrespective of location	8/12
Huang et al. 2001 [33]	Ambulance utilization in metropolitan and rural areas in Taiwan	Retrospective study	Taiwan 1997	Not specified	Patients attending an emergency medical service	Urban areas had higher call volume and non-transport calls.	5/12
Layon et al. 2003 [34]	Utstein style analysis of rural out-of-hospital cardiac arrest [OOHCA]: Total cardiopulmonary resuscitation (CPR) time inversely correlates with hospital discharge rate	Retrospective analysis	The USA 1998	65.9 ± 17.4	137 patients with cardiac arrest	Asystole as the initial rhythm, and ED CPR time (8 vs. 15.5 min, *p* = 0.042 for survivors vs. non-survivors) were the only factors affecting the survival rate.	6/12
Svensson et al. 2003 [35]	Safety and delay time in prehospital thrombolysis of acute myocardial infarction in urban and rural areas in Sweden	Prospective observational study	Sweden 1999–2000	65 ± 12/69 ± 10	154 patients with myocardial infarction	Patients in urban areas got a higher ejection fraction and fewer symptoms of heart failure after 30 days and a lower 1-year mortality	7/12
Vukmir et al. 2004 [36]	The influence of urban, suburban, or rural locale on survival from refractory prehospital cardiac arrest	Prospective, randomised, double— A blind clinical intervention trial	The USA 1992–1996	>18 years	874 patients with cardiac arrest	The survival rate was approximately 13.9% in rural, 14% in suburban, and 23% in urban	9/12
McGuffie et al. 2005 [37]	Scottish urban versus rural trauma outcome study.	Prospective observational study	Scotland 1998–2000	Urban median = 50 years, rural median = 46 years	4636 traumatic patients	Rural areas had higher transfers than urban areas (*p* = 0.002).	7/12
Gonzalez et al. 2006 [20]	Increased Mortality in Rural Vehicular Trauma: Identifying Contributing Factors Through Data Linkage	Retrospective analysis	The USA 2001–2003	Not specified	6443 patients with crash injuries	Mortality rates were 4.2% and 2.1% in rural and urban respectively (*p* = 0.0001).	6/12
Herlitz et al. 2006 [38]	Characteristics and outcome of patients with acute chest pain about the use of ambulances in an urban and a rural area	Cross-sectional study	Sweden 1996–1997	71 ± 15/59 ± 17	Patients with acute chest pain	The Mortality rate was 41.8% among those transported by ambulance and 15.8% among those transported by other means.	7/12
Jennings et al. 2006 [19]	Out-of-hospital cardiac arrest in Victoria: Rural and urban outcomes	Retrospective case series	Australia 2002 to 2003	68.4 ± 14.4/65.2 ± 13.4	1790 patients with bystander-witnessed cardiac arrest	Rural areas had more bystander cardiopulmonary resuscitation than urban areas. Urban patients with bystander-witnessed cardiac arrest were more likely to discharge from hospital alive than rural patients.	5/12
Moore et al. 2008 [39]	The Northern Ireland Public Access Defibrillation (NIPAD) study: Effectiveness in urban and rural populations	Prospective before and after the intervention, population study.	Northern Ireland 2004–2006	67.9 (15.1)	Patients with out-of-hospital cardiac arrests	In the urban areas, rates of survival were 5.1% in 2004 and 1.4% from 2005 to 2006. In the rural areas, survival rates were 2.5% in 2004 and 3.5% in 2005–2006.	8/12
Gonzalez et al. 2009 [29]	Does increased emergency medical services prehospital time affect patient mortality in rural motor vehicle crashes?	Retrospective analysis	The USA 2001–2002	Not specified	45,763 crashed patients	Rural settings had a higher mortality rate than urban settings. 1.78% in rural settings versus 0.90% in urban settings (*p* < 0.0001).	7/12
Mihalicz et al. 2010 [40]	Urban vs. rural pediatric trauma in Alberta: Where can we focus on prevention?	Retrospective analysis	USA 1996–2006	11 (0–17)	2660 paediatric patients with major trauma	Urban patients had a higher rate of mortality than rural ones (13.0% vs. 10.5%; *p* = 0.05).	8/12
Shultis et al. 2010 [41]	Striking Rural-Urban Disparities Observed in Acute Stroke Care Capacity and Services in the Pacific Northwest: Implications and Recommendations	Survey study	USA Spring 2008	≥45 years	426 patients with acute stroke	Rural-urban differences were observed, with rural hospitals have a much lower capacity to care for patients with stroke adequately.	8/12
Fatovich et al. 2011 [42]	A comparison of metropolitan vs. major rural trauma in Western Australia	Retrospective study	Australia 1997–2006	40.1 ± 22.6	3333 patients with major trauma	Rural patients had higher mortality rates than urban. The adjusted odds ratio for death was 1.10 (95% CI 0.66–1.84, *p* = 0.708).	7/12
Sørensen et al. 2011 [43]	Urban and rural implementation of pre-hospital diagnosis and direct referral for primary percutaneous coronary intervention in patients with acute ST-elevation myocardial infarction	Prospective analysis	Denmark 2004–2007	Range = 56 to 79	759 patients with myocardial infarctions	Rural areas had an EMS delay of 9 min compared to urban areas, and a median travel distance of 30 km longer.	6/12
Bhuyan et al. 2013 [44]	Rural-urban differences in acute myocardial infarction mortality: Evidence from Nebraska	Retrospective analysis	The USA 2005–2009 and 2011	15 to 85+	Patients with acute myocardial infarction	Urban areas had a lower mortality rate than patients in rural areas.	8/12
Ro et al. 2013 [45]	A trend in epidemiology and outcomes of out-of-hospital cardiac arrest by urbanization level: A nationwide observational study from 2006 to 2010 in South Korea. Resuscitation	nationwide observational study	South Korea. 2006 –2010	65 (49–76)	97291 patients with out-of-hospital cardiac arrest	The survival rate was 3.0% for EMS-assessed Out-of-Hospital Cardiac Arrests (OHCAs) (3.3% for cardiac aetiology and 2.3% for noncardiac aetiology) and 3.6% for EMS-treated OHCAs.	8/12
Aftyka et al. 2014 [22]	Are there any differences in medical emergency team interventions between rural and urban areas?	Retrospective cohort study	Poland 2009	Not specified	1624 patients in emergency medical service	Rural areas used cervical collars more than urban areas.	9/12
Horeczko et al. 2014 [46]	Urban and Rural Patterns in Emergent Pediatric Transfer: A Call for Regionalization	National survey data	The USA 1995–2010.	<18 years	283,232,058 paediatric emergency department visits	Children transferred from rural Emergency Departments (EDs) were more likely to arrive by emergency medical services than children transferred from urban EDs.	6/12
Lipsky et al. 2014 [47]	A comparison of rural versus urban trauma care	Observational cohort study	The USA 1995–1996	32.5 (Inter quartile range (IQR): 21.5 –50.5)	1122 traumatic patients	Mortality was comparable between urban and rural areas.	5/12
Sidney et al. 2014 [48]	Utilization of a State Run Public Private Emergency Transportation Service Exclusively for Childbirth: The Janani (Maternal) Express Program in Madhya Pradesh, India	Cross-sectional facility-based study	India 2012–2013	Median = 23	1126 women delivering in health facilities	Uptake was more in rural areas 44% and poorly educated women 40%	7/12
Masterson et al. 2015 [49]	Urban and rural differences in out-of-hospital cardiac arrest in Ireland	Retrospective analysis	Ireland 2012	67(52–78)	1798 patients with out-of-hospital cardiac arrests	Urban patients had higher hospital discharge rates than rural patients (6% vs. 3%)	5/12
Nordberg et al. 2015 [50]	The survival benefit of dual dispatch of EMS and fire-fighters in out-of-hospital cardiac arrest may differ depending on population density – A prospective cohort study	Prospective cohort study	Sweden 2004, 2006–2009	77/72	2513 patients with out-of-hospital cardiac arrest	30-day survival was higher in urban patients than the rural patients.	7/12
Raatiniemi et al. 2015 [51]	Short-term outcome and differences between rural and urban trauma patients treated by mobile intensive care units in Northern Finland: A retrospective analysis	Retrospective analysis	Finland 2012–2013	33 (20–55)	472 traumatic patients	Mortality within 30-day was 23.9% in urban and 13.3% in rural.	8/12
Newgard et al. 2016 [21]	Evaluation of Rural vs. Urban Trauma Patients Served by 9-1-1 Emergency Medical Services.	Secondary analysis of a prospective cohort study	The USA 2011	51.6 ± 26.1	53,487 traumatic patients	Mortality was 23.9% in urban and 13.3% in rural, however, in the first 24 h 89.6% of rural deaths occurred compared with 64% of urban deaths.	6/12
Beck et al. 2017 [52]	Resuscitation attempts and duration in the traumatic out-of-hospital cardiac arrest	Retrospective analysis	Australia 2008–2014	Median = 44 years (IQR: 28–60)	2334 patients with traumatic out-of-hospital cardiac arrest	Arrests occurring in urban regions had significantly higher odds of attempted resuscitation relative to those in rural regions	8/12
Mathiesen et al. 2018 [53]	Effects of modifiable prehospital factors on survival after out-of-hospital cardiac arrest in rural versus urban areas	Prospective analysis	Norway. 2006–2014	Urban = 70 (58–81), rural = 69 (56–80)	1138 patients with out-of-hospital cardiac arrest	Urban patients had higher survival rates than urban patients.	9/12
Park et al. 2018 [54]	Dispatcher-assisted bystander cardiopulmonary resuscitation in rural and urban areas and survival outcomes after the out-of-hospital cardiac arrest.	Cross-sectional study	South Korea 2012–2015	71 (57–79)	53,240 patients with out-of-hospital cardiac arrests	Urban patients had higher neurological recovery than rural patients. 1.6% and 6.8% in rural and urban areas, respectively.	9/12
